# Delivering Value from the Digital Integration in Medical Imaging Centers in Healthcare Organizations

**DOI:** 10.5334/jbr-btr.1362

**Published:** 2018-01-03

**Authors:** Bart L. Claikens

**Affiliations:** 1 AZ Damiaan Oostende, Department of Medical Imaging and Radiology, Ostend, BE

**Keywords:** healthcare, information technology, RIS, PACS, HIS, medical imaging, benefits dependency network

## Abstract

The mission of our person-centered and value-based health care system is to provide high-quality and safe care, being payable and accessible. In this care, the goal of health information technology (HIT) is to improve the well-being of individuals and communities through the use of new technologies and deploying a resilient health information system (HIS), infrastructure and architecture.

## Why Do We Want Improvement and Implementation of Resilient Health Information Systems?

In April 2015, the Minister of Belgian Social Affairs and Public Health, Maggie De Block, published her strategic plan for reforming the hospital financing towards improving the quality and moreover the efficiency of providing care in hospitals [1].

The strategy on people-centered and integrated health services is a call for a fundamental paradigm shift in the way health services are funded, managed and delivered. This is urgently needed to meet the challenges being faced by health systems as populations are living longer and the burden of costly long-term chronic conditions and preventable illnesses, that require multiple complex interventions over many years, continues to grow [2].

On the other hand, important financial challenges occur nowadays given the available and shrinking budgets and the rising health care spending [3]. Long-term care has risen over the past few decades and is expected to rise further in the coming years. New medical technologies are improving diagnosis and treatment but they are also increasing health spending.

Wise use of these budgets, with operational performance, and creating a culture of value based decision-making is undeniably a primary goal for current hospital managers and leading physicians.

These challenges in healthcare are based on the changing demographics, the demand for operational excellence, regulatory commitments, need for innovation and financial health [4].

## What Improvements Do We Want in the Health Information System (HIS)?

Given the shrinking budgets, not only the call for transparency and good leadership, but also sustainable and integrated information systems, new technologies and business alignment are more crucial than ever. Deep health information system integration is mandatory in order to increase efficiency. Healthcare organisations are obliged to adopt these new technologies.

This evolution increases complexity in many ways and calls for an open mind set in operational excellence, lean management, and business intelligence as a solid base to further excel in clinical and service quality.

In particular, the most crucial change in medical imaging centers in recent years has been the introduction of digital Picture Archiving and Communicating Systems (PACS) and the Radiology Information System (RIS) to deliver cost-effective solutions informing communicating to the right person in the right place and time.

## What Are the True Benefits for the Stakeholders?

Advanced integrated digital workflow solutions running through a network are helping technology-savvy imaging centers realize new efficiencies: patient registration, exam scheduling, reporting systems, image archiving and the secure connection to electronic medical patient files.

An integrated Picture Archiving and Communications System (PACS) and a Radiology Information System (RIS) empowers highly efficient workflow. RIS-driven workflow provides access to patient information (patient demographics, current and prior images, radiology results and other clinical data) through a single and secured point of entry pushed from the electronic patient data management system (EPDM). On the other hand, integration with the hospital information system (HIS) facilitates overall patient care, hospital management, research and education. The medical imaging department value chain is in its images and clinical reporting.

The result is a more efficient and lean medical workplace allowing streamlined communications with all stakeholders: referring physicians, other healthcare providers, patients and the hospital management.

A key benefit of RIS/PACS integration is the reduction or elimination of the costs of film, developing and supplies, file room space and film management (resources). The savings often offset the cost of a RIS/PACS solution.

Integrating speech recognition with RIS/PACS will also significantly decrease or even eliminate the transcription costs. One of the major advantages of all this technology for the referring physicians is that they will have access to images and reports, as soon as radiologists have read and signed off the study (time), in a secure environment (legal).

Integrating RIS/PACS with Practice Management/Electronic Medical Record (PM/EMR) solutions enhances operational efficiencies with computerized patient medical records, exam scheduling, imaging center revenue management and more (transparency). At the same time, it serves as an excellent marketing tool by opening up fast and convenient new avenues of communications with our health care environment.

In allowing remote coverage of multiple sites by the same radiologists and remote consultations and expert opinions, teleradiology is in many instances the only option to maintain economically viable radiologic settings (cross-border added values).

Based on key technology trends (like connectivity, communication, IOT, Cloud, Big data) and the key market shift (behavior of customers-patients-healthcare providers, digitalization, new ecosystems), medical imaging centers and healthcare providing organizations have to apply a digital strategy (context – ways, processes, modes – ends) to optimize the organizational and operational performance.

Based on case-based problem analysis with the key stakeholders (medical imaging departments as key-users), information technologies (PACS, RIS, PM, EMR) can bring seamless and sustainable solutions.

**Figure 1:**
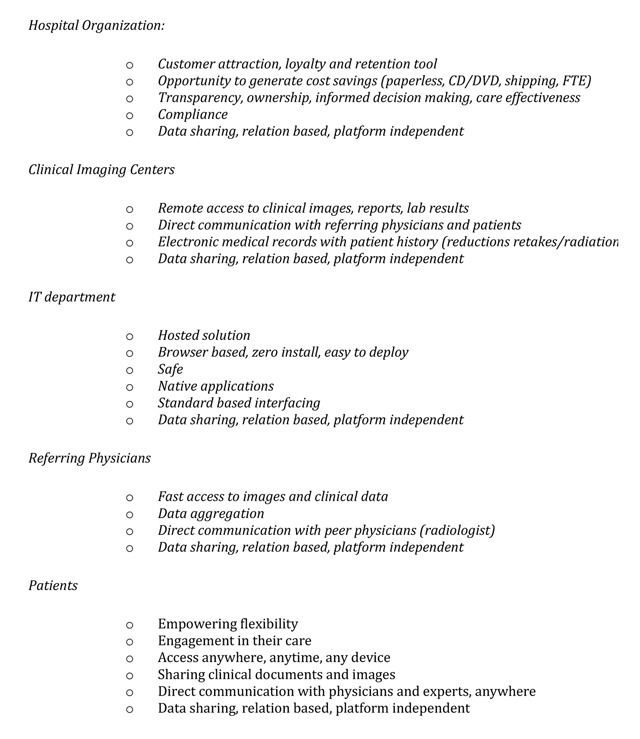
IT benefits of all the stakeholders.

A comprehensive overview of the IT benefits of all the stakeholders is shown in Figure 1.

## What about Adopting Information Systems in Healthcare and Stakeholders’ Alignment?

In comparison with other industries, health care has lagged in the adoption of information technology, contributing to gaps in clinical and business processes and systems that have hampered process improvement efforts [5].

The chasm between information system requirements and the organizational objectives of healthcare providers is often a leading cause of failure in adopting these technologies and their alignment. Requirements are often established without understanding the implications to workflow and organisational change. While stakeholder analysis provides useful insights for the *big picture*, the impact of requirements on stakeholder interests aren’t known. Similarly, low-level process modelling has trouble representing the broader organisational design and business objectives.

## The Benefits Dependency Network

The benefits dependency network (BDN) serves here as a useful and tangible model as well as a bridge between the two by tracing the impact of key technical requirements on organisational change, resultant outcomes, and benefits that accrue to stakeholders across the ecosystem – individuals, families, caregivers, health care entities, care providers, payers, technology developers, academic institutions and governments. A strong link is shown between the governance of organisational stakeholders and the dynamics of the technology introduction process.

**Figure 2:**
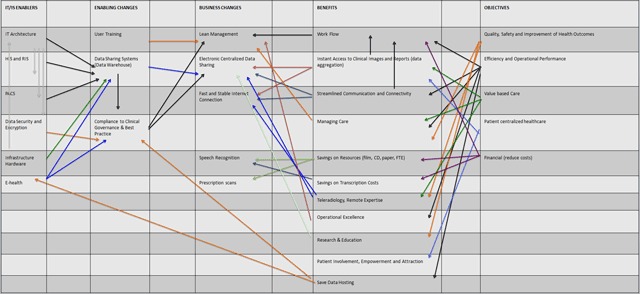
Benefits dependency network for an IT/IS investment.

Figure 2 shows the benefits dependency network for an IT/IS investment in a hospital organization with focus on the medical imaging department, revealing the organization objectives, overall benefits, business changes, enabling changes and information technology and system enablers.

This clearly demonstrates how the objectives of a hospital organization and its mission could be met, using the current available information technology, while taking into account all the stakeholders. Using this Benefit Dependency Network (BDN) model, the hospital organisation can identify the needs and benefits of all the stakeholders in order to successfully invest and implement the information technology and systems (HIS, RIS, PACS) in a sustainable way.

## Challenges for Care Providers

An unfortunate problem with health IT is the tendency to burden healthcare professionals with additional administrative responsibilities. This imposes for less time the care providers to interact with patients and other care team members. Hospital organizations need to be attentive to the unintended consequences of this trend. Dyad leadership can be the key driver to this problem. Dyad leadership, where a clinical leader is paired and aligned with and administrative leader with common values and clear roles, can build the pathways [6]. Nevertheless, the building of a benefits dependency network for an IT/IS investment is already first step to create awareness, integration and involvement throughout all key-stakeholders.

## Conclusion

This article concludes that the benefits dependency network (BDN) has utility as a very adaptive tool and an integrative framework for understanding the intertwined contributions of organizational design in hospital enterprises and the information technology and systems upon various stakeholders, particularly on medical imaging departments.

In a hospital area, technology applications currently could support financial and administrative management, clinical and patient management and data analytics. It is seen as a tool for operational excellence, patient intimacy and service leadership in our patient-centric healthcare environment.

This new model of collaborative project delivery, on the contrary, also poses challenges for all stakeholders through involvement and participation.

Modernizing and redesigning technology infrastructure is vital for advancing the health and well-being of individuals and communities across our nation, and should be available and implemented when and where it matters most.

## Author Note

In this article we focus and discuss on the benefits-based analysis of the use of information technology in hospital based medical imaging departments.
